# Home-Based HIV Testing and Counseling for Male Couples (Project Nexus): A Protocol for a Randomized Controlled Trial

**DOI:** 10.2196/resprot.7341

**Published:** 2017-05-30

**Authors:** Rob Stephenson, Ryan Freeland, Stephen P Sullivan, Erin Riley, Brent A Johnson, Jason Mitchell, Deborah McFarland, Patrick S Sullivan

**Affiliations:** ^1^ School of Nursing Department of Health Behavior and Biological Sciences University of Michigan Ann Arbor, MI United States; ^2^ The Center for Sexuality and Health Disparities University of Michigan Ann Arbor, MI United States; ^3^ University of Rochester Medical Center Department of Biostatistics and Computational Biology University of Rochester Rochester, NY United States; ^4^ Office of Public Health Studies University of Hawai'i at Mānoa Honolulu, HI United States; ^5^ Rollins School of Public Health Hubert Department of Global Health Emory University Atlanta, GA United States; ^6^ Rollins School of Public Health Department of Health Policy and Management Emory University Atlanta, GA United States; ^7^ Rollins School of Public Health Department of Epidemiology Emory University Atlanta, GA United States

**Keywords:** HIV, telemedicine, male couples

## Abstract

**Background:**

HIV prevalence remains high among men who have sex with men (MSM) in the United States, yet the majority of research has focused on MSM as individuals, not as dyads, and has discussed HIV risks primarily in the context of casual sex. Nexus is an online prevention program that combines home-based HIV testing and couples HIV testing and counseling (CHTC). It allows partners in dyadic MSM relationships to receive HIV testing and care in the comfort of their designated residence, via video-based chat. By using video-based technologies (eg, VSee video chat), male couples receive counseling and support from a remote online counselor, while testing for HIV at home.

**Objective:**

This randomized control trial (RCT) aims to examine the effects of video-based counseling combined with home-based HIV testing on couples’ management of HIV risk, formation and adherence to explicit sexual agreements, and sexual risk-taking.

**Methods:**

The research implements a prospective RCT of 400 online-recruited male couples: 200 self-reported concordant-negative couples and 200 self-reported discordant couples. Couples in the control arm will receive one or two home-based HIV self-testing kits and will be asked to report their results via the study’s website. Couples in the experimental arm will receive one or two home-based HIV self-testing kits and will conduct these tests together under the facilitation of a remotely located counselor during a prescheduled VSee-based video CHTC session. Study assessments are taken at baseline, as well as at 3- and 6-month follow-up sessions.

**Results:**

Project Nexus was launched in April 2016 and is ongoing. To date, 219 eligible couples have been enrolled and randomized.

**Conclusions:**

Combining home-based HIV testing with video-based counseling creates an opportunity to expand CHTC to male couples who (1) live outside metro areas, (2) live in rural areas without access to testing services or LGBTQ resources, or (3) feel that current clinic-based testing is not for them (eg, due to fears of discrimination associated with HIV and/or sexuality).

**Trial Registration:**

ClinicalTrials.gov NCT02335138; https://clinicaltrials.gov/ct2/show/NCT02335138 (Archived by WebCite at http://www.webcitation.org/6qHxtNIdW)

## Introduction

HIV prevalence remains high among men who have sex with men (MSM) in the United States [1]. Recent research has drawn attention to the role of male dyads in the US HIV epidemic, with primary partners identified as the source of approximately one-third [2] to two-thirds [3] of new HIV infections. Given these estimates, a significant paradigm shift in HIV prevention is needed. Efforts have traditionally focused on MSM, in particular gay-identifying men, (1) as individuals versus dyads and (2) as having been the focus of messages about HIV risks, primarily in the context of casual sex. Recent research findings have illustrated high rates of sexual risk behavior for HIV with primary and casual partners, low rates of disclosure of potentially risky episodes with casual partners to primary partners, and reduced frequency of HIV testing among male couples [4-10]. In addition, relationships may convey a misplaced sense of protection [11,12], to some degree created by the historical prevention focus on reducing numbers of sexual partners among MSM [13]. The Office of the US Global AIDS Coordinator, through dissemination of prevention guidelines for MSM in the President’s Emergency Plan for AIDS Relief-supported countries, now recommends couples HIV testing and counseling (CHTC) for male couples [14].

CHTC has been used as an HIV prevention intervention for heterosexual couples in Africa for over 20 years [15]. Labeled as a “high leverage HIV prevention intervention” by the US Centers for Disease Control and Prevention (CDC) [16], CHTC is considered an effective approach to HIV prevention among male couples. The difference between CHTC and individual HIV testing and counseling is that both partners of the male couple receive counseling and testing together at the same time [17]. During the CHTC session, the counselor learns about the couple’s relationship and provides tailored counseling and HIV prevention recommendations based on the characteristics of the couple’s relationship and their joint HIV status [17]. Through the adaptation of CHTC and the high acceptability among MSM [15,18], preliminary data from MSM in three US cities—Atlanta, Chicago, and Seattle—demonstrate the readiness of US MSM to receive and use CHTC [19,20]. Preliminary data also suggests that male couples receiving CHTC do not report increased levels of intimate partner violence (IPV) or relationship dissolution [21]. CHTC is now considered by the CDC to be an effective public health strategy and is currently being implemented in over 40 US states [17,22].

A critical aspect of CHTC involves discussing a couple’s sexual agreement. Sexual agreements refer to mutually understood rules between two partners that describe the kinds of sexual behavior that are allowed within and outside of their relationship [23]. Sexual agreements are common among male couples [5,23-30]. In CHTC, male couples discuss their sexual agreements, role-play with the counselor about how they would communicate about breaking their sexual agreement to their partner, and develop an HIV prevention plan based on their sexual agreement and couple HIV serostatus. Research regarding male couples’ sexual agreements has shown that men are less likely to practice concurrent condomless anal intercourse (CAI) if they value and commit to their agreement and if they perceive their main partner to be dependable and investing in the relationship [29,31-33]. Additionally, promoting positive relationship dynamics has the potential to reduce couples’ risk for HIV, as increased trust, communication, commitment, and social support are shown to be associated with lower odds of breaking a sexual agreement, which can ultimately reduce unique HIV risks (eg, CAI) for the couple [4].

In addition to CHTC, another HIV testing option is home-based HIV testing, which was approved by the US Food and Drug Administration in 2012 [34]. Some have argued that the lack of direct counseling with home-based testing may prevent MSM from (1) fully understanding the results, (2) adopting safer preventive strategies, and/or (3) successfully linking to care if newly HIV positive [35]. One way to address the lack of counseling for home-based HIV testing may be through the use of remote online counseling delivered through video-chat software. Online counseling offers a convenient, confidential, and user-controlled opportunity to provide support and information to individuals who otherwise may not be willing or able to access services in person. Telemedicine modalities, such as email, instant messaging, chat rooms, video conferences, and interactive media, have provided online counseling services for people suffering from disabilities, depression, and anxiety; for survivors of trauma; and for cancer treatment [36]. Although the use of telemedicine to address HIV is a fairly recent development, evidence from diverse settings suggests it is feasible, acceptable, and effective [36-38]. A number of HIV prevention interventions have been designed to deliver messages and counseling through use of the Internet [39-42]; the results indicate that increases in knowledge, self-efficacy, and motivation for behavior change can be achieved with the participant sitting remotely at their computer. Few online services, however, have been developed to facilitate HIV testing and none have been tailored for couples. Furthermore, the majority of interventions have been delivered through text-based communication, which fails to capture important verbal and visual cues (eg, tone of voice and body language) of clients.

A vital cornerstone to prevention and linkage to care is HIV testing [43]. This paper describes the protocol for a new randomized controlled trial (RCT) that combines the CDC-recommended prevention strategy of CHTC with home-based testing for male couples via video-based counseling and testing. By using video-based technologies (eg, VSee video chat), male couples receive counseling and support from a remote online counselor, while testing for HIV at home. This combination of HIV testing efforts is needed and timely because few interventions exist for male couples, especially those who live in rural areas and areas with few LGBTQ resources. This RCT aims to examine and compare the intervention’s effects on the relationship’s ability to manage HIV risk, formation and adherence to explicit sexual agreements, and sexual risk-taking between self-reported concordant-negative and self-reported discordant male couples. Participants complete surveys at baseline, 3 months, and 6 months. It is hypothesized that couples exposed to the intervention will learn communication skills and receive psychoeducation that allows them to work together on HIV prevention planning. As a result, it is hypothesized that couples exposed to the intervention will be more likely to report discussing and forming sexual agreements and be more likely to adhere to agreements, yet also more likely to disclose if they break their agreements.

The theoretical basis for intervention is the Couple’s Interdependence Theory (CIT) [44], a framework that combines both interdependence theory and communal coping perspectives and captures constructs central to the intervention. The framework guides the selection of measures of behavior change within the couple’s relationships. Through interdependence, both members of the couple will rely on themselves and on each other to reach individual- and/or couple-positive and/or couple-negative healthy behaviors. These outcomes will either improve or detract from the quality of the couple’s relationship. With this in mind, communal coping assists couples in achieving positive healthy behaviors that benefit both members of the couple as they cooperatively communicate to reach their desired goals [44]. These measures relate to the intervention in two ways. First, some aspects of communication and decision making within the partnership may influence the efficacy of the intervention; couples with more constructive communication styles may benefit from CHTC than would couples with less constructive communication styles. Second, some aspects of partnerships, such as efficacy around implementing behavioral change, may benefit more from CHTC. In this way, changes in key characteristics of the partnerships may be in the causal pathway between the intervention and the adoption of greater health-enhancing behaviors (ie, reduction in CAI outside of the relationship) within the partnership. The causal pathways are conceptualized as follows: couples exposed to the intervention package will receive opportunities to talk together about HIV, sexual health, and sexual agreements within their relationship through the assistance of a trained CHTC counselor and will have the ability to self-test for HIV as a couple at home. This may in turn impact communal coping, use of coping, and transformation of motivation. This may lead to initiation and maintenance of health-enhancing behaviors, which is conceptualized as reduction in sexual risk-taking (eg, CAI) within and outside of the relationship relative to couples who self-test for HIV at home without the presence of a counselor. In the research design, predisposing factors, outcome efficacy—the shared desire for the same outcome (ie, HIV prevention strategies)—and couple efficacy are collected separately from each member of the male couple before the HIV testing intervention is delivered, and will again be collected at 3-month and 6-month follow-up assessments.

## Methods

### Description of Trial and Intervention

#### Design

The research activities involved a blind prospective RCT of approximately 400 online-recruited male couples—200 self-reported concordant-negative couples and 200 self-reported discordant couples. Couples in the control arm will receive one or two home-based HIV self-testing kits and will be asked to report their results via the study’s website. One kit will be provided for each person who reports to have previously tested HIV-negative or unknown status; partners in serodiscordant couples who report living with HIV will not be retested. Couples in the experimental arm will receive one or two home-based HIV self-testing kits and will conduct these tests together under the facilitation of a remotely located counselor during a prescheduled VSee-based video CHTC session.

#### Participants

Participants for each male couple must meet the following eligibility criteria: (1) two men who have been in a sexual relationship with each other for more than 6 months; (2) >18 years of age; (3) both participants not having been tested for HIV in the last 3 months, or for serodiscordant couples, the negative partner not having tested for HIV in the last 6 months; (4) reporting no IPV or coercion within the last 12 months; (5) willing to have HIV test kits delivered to an address they provide; (6) have access to the Internet within their home, or the home of at least one partner; and (7) be either self-reported concordant HIV negative or self-reported HIV serodiscordant. Participants for the trial are being recruited from across the United States, with recruitment via online advertisements placed on key social media websites (eg, Facebook and Instagram) and social media sites aimed specifically at MSM (eg, Grindr and Scruff). When men click on the advertisement, they are taken to a page containing basic study information, including a short description of study activities. If they express an interest in participation, they are then taken to the study consent form and if they consent, they are directed to a short eligibility screener. Men who (1) do not consent, (2) do not meet the eligibility criteria, or (3) do not provide an email for a main partner—defined as a sexual relationship with “a man who you feel committed to above all others”—are taken to a screen thanking them for their interest. Men who are eligible to participate must provide an email address for their main partner so that they can be enrolled in the study together. Further, eligible men able to participate and that provide their partner’s email address are directed to a registration process. During the registration process, both partners provide their contact information, including an email address, a mobile phone number, and a mailing address; they are also asked to provide a nickname or preferred name of choice. Once both partners have (1) completed the consent forms, (2) completed the screening questionnaire, (3) proven eligible for the study, and (4) registered on the study website, a joint email is sent to both partners asking them to complete the baseline questionnaire, individually.

#### Randomization

Upon individual completion of the baseline survey by both partners, couples are randomized to either the home-based HIV testing arm or video-based CHTC with home-based HIV testing arm using a stratified 1:1 treatment allocation. The strata are based on two levels of serostatus: seroconcordant negative and serodiscordant. The treatment assignments are generated with the use of a pseudo-random number generator with permutated blocks that are used to ensure balance within stratum between the numbers of couples assigned to each treatment. The randomization process generates one of two emails to both study participants for the enrolled male couple, indicating whether they will be receiving home-based HIV testing or video-based CHTC with home-based HIV testing.

#### Intervention

The proposed intervention is a combination of home-based HIV testing and CHTC offered remotely via VSee video chat. In the control arm, couples receive one or two home-based HIV testing kits based on the couple’s serostatus; the partner living with HIV in a serodiscordant couple does not repeat HIV testing. These kits sent to couples in the control arm contain one or two HIV testing kits as well as instructions on how to use the kits and how to report their test results in the study portal. In the experimental arm, couples receive one or two home-based HIV testing kits and complete the testing while undergoing a remote, VSee video-based CHTC session. Individual participants testing positive in either the experimental or the control condition are linked to their preference of local HIV care-specific resources within 48 hours. They will receive another HIV test at their preferred local HIV care-specific resource to validate the preliminary positive test result. These participants will receive follow-up from the CHTC counselor at 1-week, 1-month, and 3-month postresult of HIV-positive status to assess their engagement in care.

**Figure 1:**
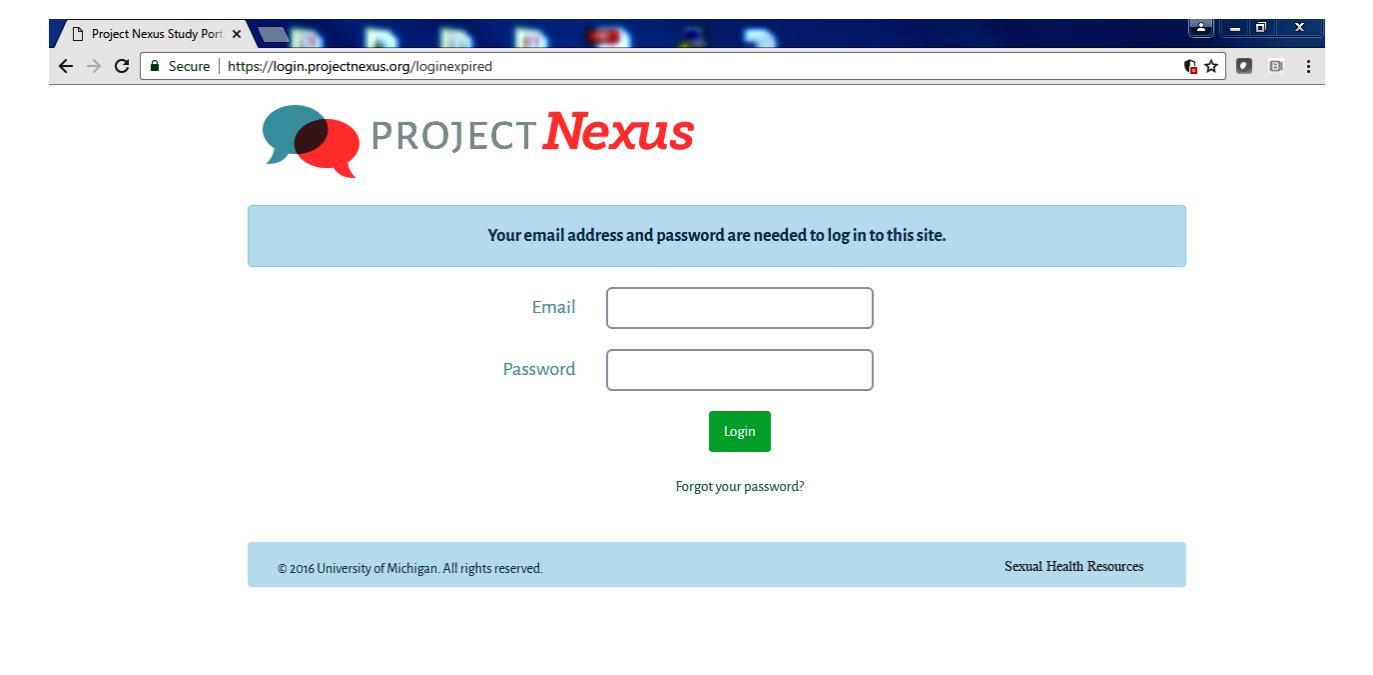
Project Nexus website log-in page.

**Figure 2:**
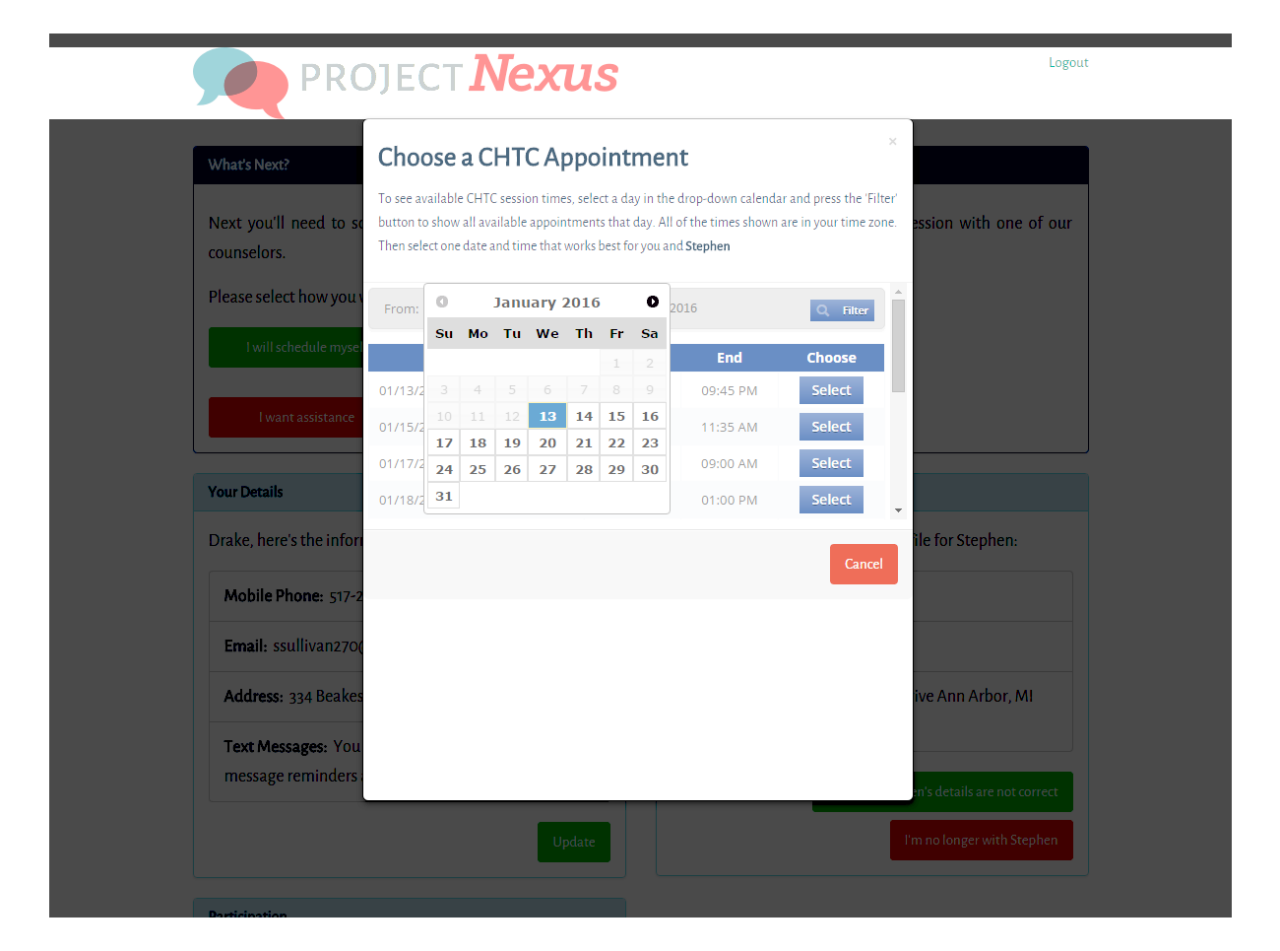
Calendar function for selecting a couples HIV testing and counseling (CHTC) appointment.

For couples randomized to the experimental arm, an email informs them that they have the opportunity to receive one or two home-based HIV testing kits and to take part in a video-based counseling session. The email provides details on the expected content of a CHTC session, the expectation that both partners will need to conduct their individual HIV tests and receive their results together in the presence of a remote counselor, as well as further logistical information (ie, length of the counseling session). From this email, couples are instructed to log on to the study website to order their HIV testing kits, with the same options as the control arm. Figure 1 shows the log-in page that users see when logging on to the Project Nexus website. For those randomized to receive HIV home testing with CHTC, they are asked to select a CHTC appointment time via an electronic calendar, which is shown in Figure 2. To allow intent-to-treat analysis, couples who do not complete the CHTC session and do not schedule an appointment, as well as couples in the control arm who do not report their HIV test results, are able to move on to take the 3-month and 6-month surveys.

**Figure 3:**
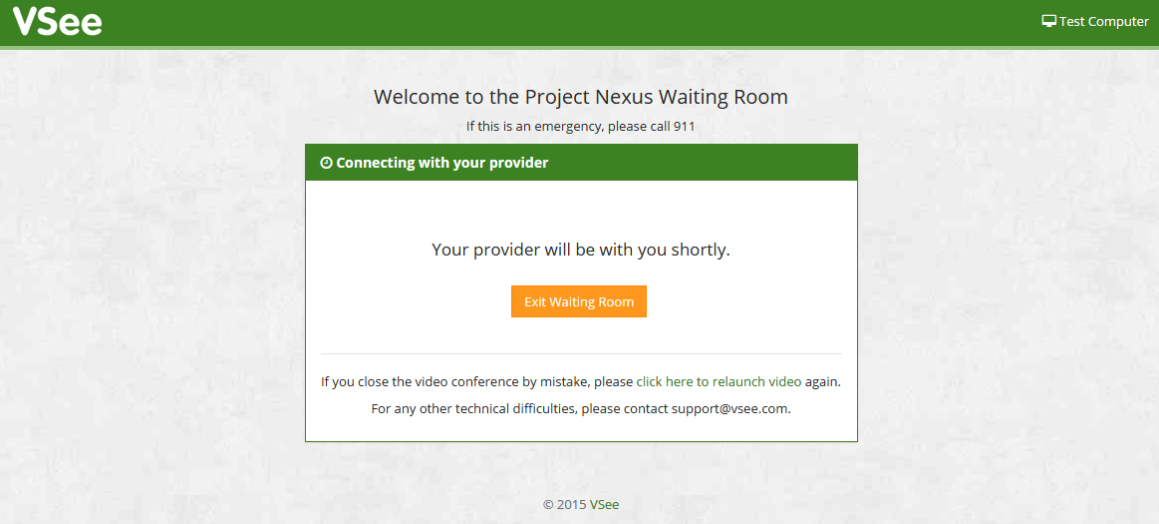
VSee session interface.

**Figure 4:**
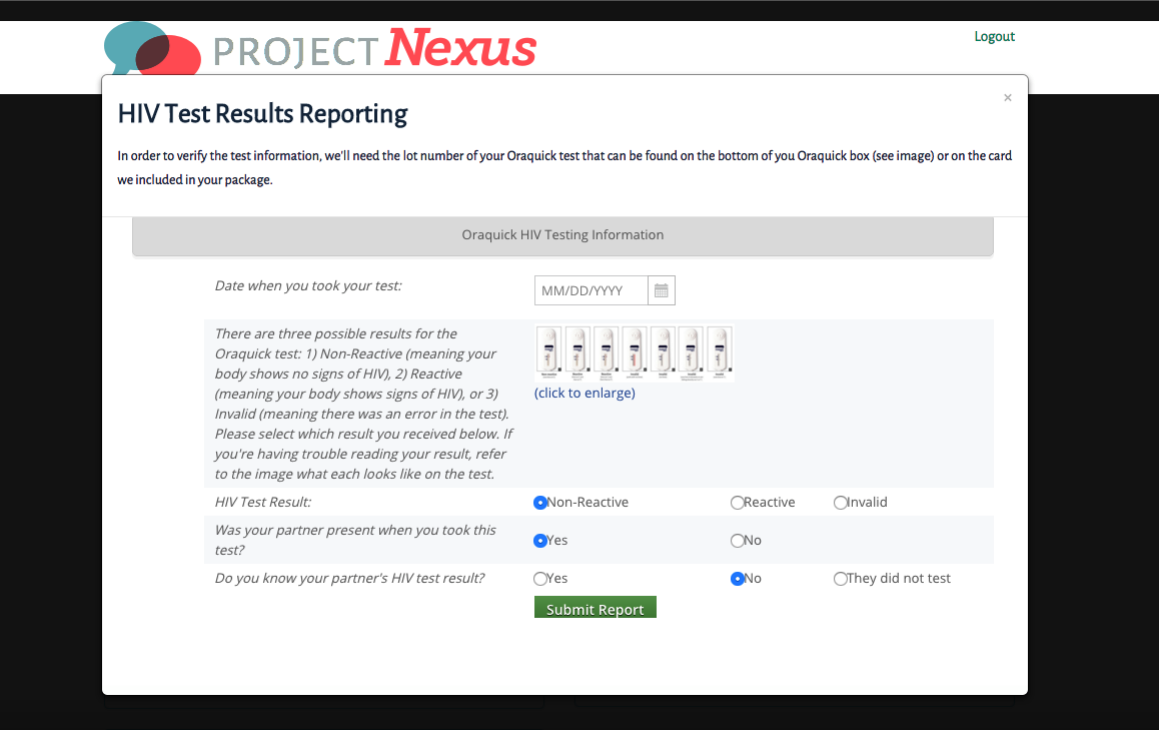
HIV test results reporting page on Nexus website.

The CHTC session is conducted via video chat using VSee. Figure 3 is an example of the VSee interface that participants see when waiting for their prescheduled counseling session to begin. The session is conducted by a counselor who is trained in CHTC and will last approximately 30-45 minutes. Pretest counseling focuses on the couple’s relationship, their perceived HIV risk factors, and focuses on their sexual agreement. Both partners individually conduct their own HIV test and read their individual results together, as instructed by the counselor. Participants are asked to show the counselor their test result; the counselor then confirms the test result for accuracy of interpretation. Figure 4 is the interface the participant sees when entering their HIV testing result to the Nexus website. Posttest counseling focuses on dyadic prevention messages and revisits the couple’s HIV risk concerns and sexual agreements in light of their test results. The counselor records the couple’s HIV test results in the couple’s study file. The counselor is trained to keep focus on the couple in the event that an HIV-positive result is given during a CHTC session. While a focus on the immediate needs of the HIV-positive partner is required, the discussion remains focused on how the couple can work together to keep the positive partner healthy and to keep the risk of HIV within, and not outside of, the relationship with other partners, while also meeting the needs of both partners within the male couple. If both couples are seroconcordant negative, the counselor discusses strategies that will minimize the risk of HIV transmission within and/or outside the relationship. The prevention-counseling element of the CHTC session focuses on talking the couple through prevention options and asking them to consider which prevention options may work best based on their relationship needs, context, and unique risk profile.

### Preliminary Data

An online survey of 1285 MSM from across the United States was conducted to examine willingness to use the proposed intervention (unpublished data). Respondents were recruited through targeted advertisements on Facebook over 10 days from October to November 2012. Of the 907 men who self-reported a negative HIV serostatus and provided complete data, 72.0% (653/907) reported they would be likely to use a home-based HIV test, 69.0% (626/907) reported they would be likely to use CHTC, and 75.0% (680/907) reported that they would be likely to use a home-based HIV test together with a VSee video-based CHTC session. The willingness to use the proposed intervention did not vary by age (*P*=.60), race (*P*=.91), recent (ie, <12 months) HIV testing behavior (*P*=.43), or recent CAI (*P*=.39). Among those with main partners (510/907, 56.2%), 82.0% (418/510) reported they would likely use the intervention. Among those who reported recent physical violence from an intimate partner (55/502, 11.0%), 67% (37/55) reported they would likely use the intervention. Emory University Center for AIDS Research also funded a trial of Skype-based counseling with 15 male couples from Atlanta to examine feasibility and acceptability of the video-chat counseling session. Although the trial did not incorporate HIV testing, 15 male couples were recruited using the same online recruitment methods as proposed in this intervention. Couples completed a baseline survey describing recent sexual risks and sexual agreements in their relationships; then each couple underwent an approximately 45-minute counseling session via Skype video-based chat. During the session, the Emory-based counselor talked to the couple about their sexual agreements and their management of HIV risks, and then referred them to local services. Approximately half of the couples were in monogamous relationships; the mean relationship duration was 17 months. A total of 10 couples had formalized agreements around sex outside of the relationship, which ranged from monogamy to allowing sex with outside partners under certain conditions. Couples then completed an online survey about their experiences with the Skype video-based counseling session. Satisfaction was universally high—mean satisfaction was 4.8 out of 5 and 100% of participants said they would recommend the service to other male couples. Crucially, 100% of couples stated that they would be willing to include HIV testing in the video-based counseling session, with the main reasons being “convenience” and “having access to counseling while testing at home.”

### Outcomes: Overview

The study outcomes are based on the conceptual model of CIT [44]. All measures are collected via the baseline, 3-month, and 6-month online surveys. Primary outcomes are the initiation and maintenance of health-enhancing behaviors. Secondary outcomes measure relationship characteristics.

### Primary Outcomes

Initiation and maintenance of health-enhancing behaviors are conceptualized as including two sets of outcomes: sexual agreements and sexual risk-taking as well as linkage and retention to HIV care.

#### Sexual Agreements

All surveys include questions taken from previous studies of male couples’ sexual agreements [45]. Participants are asked which of the following best describes their current sexual agreement with their main partner: “neither of us can have sex with outside partners,” “we can have sex with outside partners, without any conditions or restrictions,” “we can have sex with outside partners, but with conditions or restrictions,” or “we do not have an agreement.” Additional items about agreements will further assess whether couples permit (or do not permit) that certain sexual behaviors, mainly CAI and oral sex, can occur with outside partners. In follow-up surveys, participants are asked whether their agreement had been broken in the previous 3 months, if they disclosed the agreement breakage to their partner, whether they have changed their agreement, and, if relevant, how their agreement had changed.

#### Sexual Risk-Taking

Behavioral measures adapted from the National HIV Behavioral Surveillance behavioral inventory, as well as from studies using behavioral measures among thousands of MSM [46,47], will collect information both on sexual behaviors with the main sex partner in the 3 months before the interview and on sexual behaviors with all sex partners outside the relationship that may exist. For sex with the main partner, men are asked to estimate the number of anal sex acts with the main partner and the number of those acts that were condom protected. For outside the relationship, men are asked a series of questions about each outside partner. Questions include HIV status of that partner (if known), whether the sex outside the relationship was disclosed to the main partner, the number and type of sex acts (ie, oral, anal, both, etc) with each outside partner, and the proportion of those sex acts that were protected by condoms. Data on use of and adherence to preexposure prophylaxis (PrEP) are collected.

#### Linkage to HIV Care

The following outcomes as indicators of linkage to care, per the recent recommendations of the Institute of Medicine [48], are measured within 3 months of HIV diagnosis via self-report [49,50]: (1) attending at least one clinical care appointment, (2) having at least one CD4 test performed, and (3) having at least one viral load test performed.

### Secondary Outcomes: Dyadic Characteristics

#### Overview

The four elements of Lewis’ model [44]—Predisposing factors of couple, Partner’s transformation of motivation, Process of communal coping, and Use of communal coping—are referred to as dyadic characteristics. In a recent RCT of CHTC, scales were developed to capture these constructs; all scales showed strong reliability and evidence for construct validity was obtained for all scales [51]. In this intervention, each of the scales is collected in the baseline and follow-up surveys.

#### Predisposing Factors of Couple

Several scales are used to measure this element. The Perceived Severity of HIV Scale involves the perception of the personal, psychosocial, and physical consequences of a particular health threat. A total of 13 items were developed that crossed the three pertinent consequences of a particular health threat: personal, psychosocial, and physical. The Preferences for General Lifestyle Outcomes Scale is defined as the degree to which interacting partners agree about the shared or joint outcomes in their relationship and is composed of two subscales: the Preferences for General Lifestyle Scale and the Preferences for Sexual Health Outcomes Scale. The Preferences for General Lifestyle Scale includes six outcomes, including diet, nutrition, and social activities. The Preferences for Sexual Health Outcomes Scale relates to sexual health, for example, reducing one’s risk for HIV. In addition, scales to measure other predisposing factors of couples are proposed for inclusion. Conflict style determines how respondents typically handle conflict in their relationships, so the Conflict Style Inventory will be included [52]. Communication style will be measured with the Communication Patterns Questionnaire Constructive Communication subscale [53]. Finally, problem-solving skills are measured with the Adherence Problem Solving/Readiness Scale [54].

#### Partner’s Transformation of Motivation

In a recent RCT of CHTC, two measures were developed: ability of the participants to respond (1) cognitively and (2) emotionally to the health threat [21]. The scale for emotional response includes whether the respondent reports being fearful, nervous, or anxious about HIV. The scale for cognitive response includes whether the respondent reports understanding the risks of HIV transmission associated with being in a serodiscordant relationship and the risks associated with outside sex partners.

#### Process and Use of Communal Coping

Several scales are also used to measure this element. The Outcome Efficacy to Reduce HIV Threat Scale discusses how communal coping involves couples working together and making decisions together to reduce the health threat. Three subscales were created to capture the full range of outcome efficacy related to these three processes of communal coping. For the first subscale, Joint Effort, the stem “My partner and I believe that ‘working together’ versus on our own is an effective strategy” is used. For the second subscale, Communication, the stem “Communicating with my partner is an effective strategy for...” is used. For the third subscale, Planning and Decision Making, the stem “My partner and I making decisions together rather than separately is an effective strategy” is used. The items for each of the three subscales were the same as the items used for the Preference for Sexual Health Outcomes Scale. The Couple Efficacy to Reduce HIV Threat Scale defines couple efficacy as a couple’s confidence that together they can engage in communal coping efforts.

In addition to the outcomes tied to Lewis’ framework [44], we also include two additional outcomes: violence and relationship dissolution. The Conflict Tactics Scale Revised [55] assesses both perpetration and experience of IPV. Relationship dissolution is assessed using an item that asks each partner in the couple to report the current status of their relationship and reason for dissolution at follow-ups.

### Statistical Analysis: Dyadic Characteristics

#### Overview

Dyadic characteristics will be analyzed within and between couples over time by HIV status and study arm (control vs experimental). Simple *t* tests and chi-square tests will be used to investigate systematic differences among average scales between experimental and control arms and between serodiscordant and seroconcordant-negative couples. Next, regression, generalized linear mixed models, and marginal models via generalized estimating equations (GEE) will be used to model longitudinal item and scale measures. In general, participants will be nested within couples, so the participant is regarded as the experimental unit; outcomes within couples and over time are potentially correlated. Initially, an unstructured serial correlation structure for the within-subject covariance and a single correlation parameter to describe the within-couple correlation will be assumed. If the unstructured covariance structure is too weak leading to nonconvergence issues and weak identification, more structure will be placed on the residuals and stronger models will be considered, such as an exchangeable or autoregressive correlation structure. Statistical inference will be likelihood based for the mixed models while generalized score and Wald tests will be used for statistical inference in the marginal models. In the presence of missing data, both mixed and marginal models accommodate missing data (eg, dropout), although the former under a weaker (missing at random) assumption.

#### Sexual Risk-Taking

The definition of at-risk sex will be serostatus specific. For serodiscordant couples, at-risk sex will be CAI with either their main or outside partners. For seroconcordant-negative and seroconcordant-positive couples, at-risk sex will be CAI with outside sex partners. While data on PrEP use and adherence will be collected, the definition of at-risk sex focuses on condom use given the CDC recommendation for continued condom use for those adopting PrEP. The incidence of at-risk sex acts will be calculated as an incidence density, with the numerator being the number of individual at-risk sex acts and the denominator being person-years of follow-up time. Comparisons of the incidence of at-risk sex acts will be made by comparing incidence densities between the two arms. Incidence rates per couple-year of follow-up will be estimated and compared using exact methods based on the Poisson distribution by using the GEE approach. Baseline covariates include race, age, and duration of relationship. Period incidence rates—6-monthly incidence density rates—of at-risk sex will be estimated by performing a GEE Poisson regression analysis of the 6-monthly counts. This will be implemented using the PROC GENMOD procedure by SAS (SAS Institute Inc) [56] and using an exchangeable correlation structure for the repeated observations of couples. The incidence density ratio, or incidence rate ratio (IRR), is the ratio of the incidence density in one treatment group (intervention arm) to that of another group (control arm). Results by each baseline covariate will be summarized as the IRR and the 95% confidence interval. These analyses will be descriptive and include analyses stratified by race/ethnicity. Prevalence of each outcome will be calculated and prevalence of outcomes will be compared in the control and intervention groups using chi-square tests or Fisher’s exact tests, as appropriate.

#### Sexual Agreements

Separately tabulated data about disclosure of sex outside the relationship and percentage of couples with agreements involving sex outside the primary relationship will be developed. These analyses will be descriptive and include analyses stratified by race/ethnicity, study arm, and couple serostatus. These analyses will also characterize the prevalence of agreements about sex outside relationships, the extent to which those agreements are adhered, and any shift of adopting safer sexual behaviors with outside partners, again stratified by race/ethnicity and study arm. A focus of the analysis will be identifying differences across the study arms in the percentages of couples who report a shift to safer sexual agreements at follow-up. In addition, the Actor-Partner Interdependence Model (APIM), which uses the dyad (eg, couple) as the unit of analysis, will be used to predict how an individual and his partner’s reports of dyadic characteristics—described within Lewis’ model [44]—affect whether the individual formed, changed, and kept the sexual agreement he had with his main partner [31]. Procedures for using SAS to calculate actor-partner effects with dichotomous outcomes with male couples (eg, indistinguishable dyads) have previously been reported in detail [31] and will be used for the proposed RCT.

#### Linkage to Care

The percentage of HIV-positive respondents who receive a timely (<3 months) comprehensive visit—a visit including a CD4 count, viral load count, and the date of their first care visit—will be tested for significance across the two study arms. The percentages of couples who are seroconcordant negative, seroconcordant positive, and serodiscordant, as part of the description of the analysis samples, will be recorded. To assess whether the concordancy of the couple modifies the intervention effects, formal statistical tests—likelihood ratio tests in mixed models and generalized score tests in marginal models—of the null hypothesis will be conducted. The null hypothesis is that the two-way interaction effect for the aforementioned items and scales between intervention and concordancy is zero. If the statistical test does not reject the null hypothesis, the concordancy main effect in the model will be retained, removing the two-way interaction, and conclude that there is not sufficient evidence to suggest that concordancy modifies the intervention effect.

The safety of the intervention at both the individual and couple levels will be evaluated by examining reported IPV within the relationship and relationship dissolution. At the individual level, prevalence of each individual adverse outcome or any adverse outcome will be calculated. Prevalence of outcomes will be compared across the control and intervention arms, by serostatus of the couple, and by relationship duration using chi-square tests or Fisher’s exact tests, as appropriate. At the couple level, the APIM will be used to predict how an individual and his partner’s reports of dyadic characteristics affect the individual’s experience of IPV and reported relationship dissolution.

### Feasibility

In addition to the outcomes, the study will assess feasibility by examining (1) time to recruit 400 couples to the intervention and (2) rate of recruitment per 100 men expressing interest in participation. Acceptability of the intervention will be determined by analysis of data from the satisfaction survey on the intervention’s acceptability to couples. In addition, the percentages of male couples who do not complete the home-testing profile and the percentage of those not returning test results via the study website will be analyzed.

### Cost Analysis

To examine the cost of the proposed intervention, cost data are collected by input type and by activity, using an activity-based costing matrix. Input types include study personnel salaries and benefits, supplies, equipment, HIV testing kits, and training materials. Activities include training (ie, counselors and crisis counseling), recruitment of participants, VSee video-based chat, follow-up, and linkage to care. For the experimental arm, counselors record the time spent per session of the counselor because these are the only additional (ie, incremental) costs incurred relative to the control arm.

### Incentives

Individual participants each receive US $50 for completing each of the main three surveys: the baseline, the 3-month follow-up, and the 6-month follow-up. If all surveys are completed by both members of the male couple, the total incentive amount is US $300 per couple (US $150 per individual participant).

### Sample Size

The goal is to enroll and maintain a sample of 400 male couples: 200 serodiscordant and 200 seroconcordant couples, 100 of each couple per arm (experimental vs control). To achieve this, the intervention aims to screen approximately 1000 male couples—500 serodiscordant and 500 seroconcordant male couples—and will exclude those with a recent (<12 months) history of IPV. Assuming 15% of male couples have a recent history of IPV, this will produce approximately 850 couples for randomization, 425 per arm. Allowing for 20% loss to follow-up and additional 20% relationship dissolution, this will produce a sample of 400 male couples who are expected to complete the prospective RCT. The sample size is calculated based on the detection of significant changes in each of the main outcomes (ie, changes in sexual risk-taking, such as CAI), formation and adherence to explicit sexual agreements, and relationship functioning for the management of HIV risk. As an example, we will assume that about 25% of male couples change agreement status after couples counseling and we will use a two-sample test of binomial proportions with type I error rate at 5%. Using these assumptions, it is determined that 52%, 67%, and 85% statistical power is necessary to detect a difference of 10%, 12%, and 15%, respectively, in a change-of-agreement status between the two arms within seroconcordant or serodiscordant groups (ie, comparing 150 male couples per arm). In other words, under the same conditions, the probability is .52, .67, and .85 that the lower bound of the confidence interval for the proportion change in agreement status in the intervention arm is at least 3%, 5%, and 8% different than the proportion in the control arm, respectively, when the alternative hypothesis is true. If data is pooled across seroconcordant and serodiscordant couples, assuming that combining data makes sense in terms of the magnitude and direction of the intervention effect, then 81%, 92%, and 99% statistical power is necessary to observe differences of 10%, 12%, and 15%, respectively (ie, comparing 300 male couples per arm). In this case, at least 80% statistical power will be necessary to detect as little as a 5% difference between the lower bound of the confidence interval for the proportion in the intervention versus the control arm. Based on discussions with participant advocates and partnered community-based organizations, it is projected that any difference exceeding 5% would be scientifically meaningful and would have public health impact. The analysis also considers differences in linkage to HIV care among HIV-positive individuals in each arm, but it is not powered to detect significant differences, given the small number of incident positive cases.

### Trial Registration, Ethics, Consent, and Institutional Board Approval

The research and ethics presented in this study have been reviewed and approved by the University of Michigan Institutional Review Board (HUM00102906), in addition to the Data Safety Monitoring Board. The study is also registered on ClinicalTrials.gov (NCT02335138).

## Results

Project Nexus was launched in April 2016 and is ongoing. To date, 219 couples have been enrolled and randomized, with 219 couples remaining eligible for continued study participation, as they have reportedly not experienced IPV, ended a relationship, falsified information, or any other criteria that would make them ineligible for study participation. Of all eligible couples, 95% have taken the baseline survey; the remaining couples are all within the time between enrollment and taking the survey. The 3- and 6-month follow-up surveys have maintained high retention rates at over 90%. Of couples randomized to the control arm, 88.0% (95/108) have reported their home HIV testing results. Of couples randomized to the intervention arm, 76.6% (85/111) have scheduled and completed the video-chat-based counseling session. In total, 5.9% (26/438) of participants have tested preliminarily as HIV positive, of which 73% (19/26) were actively linked to care.

## Discussion

Online CHTC via video chat provides an opportunity to expand CHTC to male couples who (1) live outside metro areas, (2) live in rural areas without access to testing services or LGBTQ resources, or (3) feel that current clinic-based testing is not for them (eg, due to fears of discrimination associated with HIV and sexuality). Although home-based HIV testing is now a reality, many still question the lack of counseling available to those who are undergoing testing in the individual household. A video-chat-based CHTC session potentially provides an inexpensive way to remedy this problem and provides an opportunity for those receiving an HIV-positive result to receive assistance in linkage to care. The proposed activities not only have the potential to expand CHTC to male couples who currently do not have access, but may provide opportunities to improve the utility of home-based testing for couples by providing them a forum to discuss prevention planning with a counselor. It is true that some individuals use home-based HIV testing because they do not want counseling and the proposed intervention would probably not be adopted by those people. However, for couples who desire and/or need counseling and do not have access due to physical or sociocultural barriers, a low-cost, video-based counseling session provides the opportunity to reach them, create HIV prevention planning, and locate linkage to care in a safe and comfortable environment.

## Abbreviations

APIM: Actor-Partner Interdependence Model
CAI: condomless anal intercourse
CDC: US Centers for Disease Control and Prevention
CHTC: couples HIV testing and counseling
CIT: Couple’s Interdependence Theory
GEE: generalized estimating equations
IPV: intimate partner violence
IRR: incidence rate ratio
MSM: men who have sex with men
PrEP: preexposure prophylaxis
RCT: randomized controlled trial

## References

[1] Sullivan PS, Wolitski RJ, Wolitski RJ, Stall R, Valdiserri RO (2008). HIV infection among gay and bisexual men. Unequal Opportunity: Health Disparities Affecting Gay and Bisexual Men in the United States.

[2] Goodreau SM, Carnegie NB, Vittinghoff E, Lama JR, Sanchez J, Grinsztejn B, Koblin BA, Mayer KH, Buchbinder SP (2012). What drives the US and Peruvian HIV epidemics in men who have sex with men (MSM)?. PLoS One.

[3] Sullivan PS, Salazar L, Buchbinder S, Sanchez TH (2009). Estimating the proportion of HIV transmissions from main sex partners among men who have sex with men in five US cities. AIDS.

[4] Gomez AM, Beougher SC, Chakravarty D, Neilands TB, Mandic CG, Darbes LA, Hoff CC (2012). Relationship dynamics as predictors of broken agreements about outside sexual partners: Implications for HIV prevention among gay couples. AIDS Behav.

[5] Hoff CC, Beougher SC, Chakravarty D, Darbes LA, Neilands TB (2010). Relationship characteristics and motivations behind agreements among gay male couples: Differences by agreement type and couple serostatus. AIDS Care.

[6] Stephenson R, White D, Darbes L, Hoff C, Sullivan P (2015). HIV testing behaviors and perceptions of risk of HIV infection among MSM with main partners. AIDS Behav.

[7] Stall R, Mills TC, Williamson J, Hart T, Greenwood G, Paul J, Pollack L, Binson D, Osmond D, Catania JA (2003). Association of co-occurring psychosocial health problems and increased vulnerability to HIV/AIDS among urban men who have sex with men. Am J Public Health.

[8] Chakravarty D, Hoff CC, Neilands TB, Darbes LA (2012). Rates of testing for HIV in the presence of serodiscordant UAI among HIV-negative gay men in committed relationships. AIDS Behav.

[9] Mitchell JW, Petroll AE (2012). Patterns of HIV and sexually transmitted infection testing among men who have sex with men couples in the United States. Sex Transm Dis.

[10] Phillips G, Hightow-Weidman LB, Arya M, Fields SD, Halpern-Felsher B, Outlaw AY, Wohl AR, Hidalgo J (2012). HIV testing behaviors of a cohort of HIV-positive racial/ethnic minority YMSM. AIDS Behav.

[11] Pruitt KL, White D, Mitchell JW, Stephenson R (2015). Sexual agreements and intimate-partner violence among male couples. Int J Sex Health.

[12] Goldenberg T, Clarke D, Stephenson R (2013). “Working together to reach a goal”: MSM's perceptions of dyadic HIV care for same-sex male couples. J Acquir Immune Defic Syndr.

[13] Kelly JA, St Lawrence JS, Diaz YE, Stevenson LY, Hauth AC, Brasfield TL, Kalichman SC, Smith JE, Andrew ME (1991). HIV risk behavior reduction following intervention with key opinion leaders of population: An experimental analysis. Am J Public Health.

[14] (2011). The US President's Emergency Plan for AIDS Relief (PEPFAR): Technical Guidance on Combination HIV Prevention.

[15] Sullivan PS, Stephenson R, Grazter B, Wingood G, Diclemente R, Allen S, Hoff C, Salazar L, Scales L, Montgomery J, Schwartz A, Barnes J, Grabbe K (2014). Adaptation of the African couples HIV testing and counseling model for men who have sex with men in the United States: An application of the ADAPT-ITT framework. Springerplus.

[16] Painter TM (2001). Voluntary counseling and testing for couples: A high-leverage intervention for HIV/AIDS prevention in sub-Saharan Africa. Soc Sci Med.

[17] Bazzi AR, Fergus KB, Stephenson R, Finneran CA, Coffey-Esquivel J, Hidalgo MA, Hoehnle S, Sullivan PS, Garofalo R, Mimiaga MJ (2016). A dyadic behavioral intervention to optimize same sex male couples' engagement across the HIV care continuum: Development of and protocol for an innovative couples-based approach (Partner Steps). JMIR Res Protoc.

[18] Wagenaar Bradley H, Christiansen-Lindquist Lauren, Khosropour Christine, Salazar Laura F, Benbow Nanette, Prachand Nik, Sineath R Craig, Stephenson Rob, Sullivan Patrick S (2012). Willingness of US men who have sex with men (MSM) to participate in Couples HIV Voluntary Counseling and Testing (CVCT). PLoS One.

[19] Stephenson R, Sullivan PS, Salazar LF, Gratzer B, Allen S, Seelbach E (2011). Attitudes towards couples-based HIV testing among MSM in three US cities. AIDS Behav.

[20] Neme S, Goldenberg T, Stekler JD, Sullivan PS, Stephenson R (2015). Attitudes towards couples HIV testing and counseling among Latino men who have sex with men in the Seattle area. AIDS Care.

[21] Sullivan PS, White D, Rosenberg ES, Barnes J, Jones J, Dasgupta S, O'Hara B, Scales L, Salazar LF, Wingood G, DiClemente R, Wall KM, Hoff C, Gratzer B, Allen S, Stephenson R (2014). Safety and acceptability of couples HIV testing and counseling for US men who have sex with men: A randomized prevention study. J Int Assoc Provid AIDS Care.

[22] Stephenson R, Grabbe KL, Sidibe T, McWilliams A, Sullivan PS (2016). Technical assistance needs for successful implementation of couples HIV testing and counseling (CHTC) intervention for male couples at US HIV testing sites. AIDS Behav.

[23] Hoff CC, Beougher SC (2010). Sexual agreements among gay male couples. Arch Sex Behav.

[24] Crawford JM, Rodden P, Kippax S, Van de Ven P (2001). Negotiated safety and other agreements between men in relationships: Risk practice redefined. Int J STD AIDS.

[25] Gass K, Hoff CC, Stephenson R, Sullivan PS (2012). Sexual agreements in the partnerships of Internet-using men who have sex with men. AIDS Care.

[26] Hosking W (2014). Australian gay men's satisfaction with sexual agreements: The roles of relationship quality, jealousy, and monogamy attitudes. Arch Sex Behav.

[27] Kippax S, Noble J, Prestage G, Crawford JM, Campbell D, Baxter D, Cooper D (1997). Sexual negotiation in the AIDS era: Negotiated safety revisited. AIDS.

[28] Mitchell JW (2014). Characteristics and allowed behaviors of gay male couples' sexual agreements. J Sex Res.

[29] Mitchell JW, Harvey SM, Champeau D, Moskowitz DA, Seal DW (2012). Relationship factors associated with gay male couples' concordance on aspects of their sexual agreements: Establishment, type, and adherence. AIDS Behav.

[30] Prestage G, Jin F, Zablotska I, Grulich A, Imrie J, Kaldor J, Honnor G, Kippax S (2008). Trends in agreements between regular partners among gay men in Sydney, Melbourne and Brisbane, Australia. AIDS Behav.

[31] Mitchell JW, Champeau D, Harvey SM (2013). Actor-partner effects of demographic and relationship factors associated with HIV risk within gay male couples. Arch Sex Behav.

[32] Mitchell JW, Garcia L, Champeau D, Harvey SM, Petroll AE (2012). HIV-negative seroconcordant gay male couples' attitudes, intentions, and perceived behavioral control for planned condom use within and outside of their relationships. Int J Sex Health.

[33] Mitchell JW, Harvey SM, Champeau D, Seal DW (2012). Relationship factors associated with HIV risk among a sample of gay male couples. AIDS Behav.

[34] (2014). US Food & Drug Administration.

[35] Wood BR, Ballenger C, Stekler JD (2014). Arguments for and against HIV self-testing. HIV AIDS (Auckl).

[36] Mallen MJ, Vogel DL, Rochlen AB, Day SX (2005). Online counseling: Reviewing the literature from a counseling psychology framework. Couns Psychol.

[37] Hottes TS, Farrell J, Bondyra M, Haag D, Shoveller J, Gilbert M (2012). Internet-based HIV and sexually transmitted infection testing in British Columbia, Canada: Opinions and expectations of prospective clients. J Med Internet Res.

[38] Moskowitz DA, Melton D, Owczarzak J (2009). PowerON: The use of instant message counseling and the Internet to facilitate HIV/STD education and prevention. Patient Educ Couns.

[39] Shoveller J, Knight R, Davis W, Gilbert M, Ogilvie G (2012). Online sexual health services: Examining youth's perspectives. Can J Public Health.

[40] Bowen AM, Williams ML, Daniel CM, Clayton S (2008). Internet based HIV prevention research targeting rural MSM: Feasibility, acceptability, and preliminary efficacy. J Behav Med.

[41] Chiasson MA, Hirshfield S, Rietmeijer C (2010). HIV prevention and care in the digital age. J Acquir Immune Defic Syndr.

[42] DeGuzman MA, Ross MW (1999). Assessing the application of HIV and AIDS related education and counselling on the Internet. Patient Educ Couns.

[43] Office of National AIDS Policy (ONAP) (2015). National HIV/AIDS Strategy for the United States: Updated to 2020.

[44] Lewis MA, McBride CM, Pollak KI, Puleo E, Butterfield RM, Emmons KM (2006). Understanding health behavior change among couples: An interdependence and communal coping approach. Soc Sci Med.

[45] Mitchell Jason W (2014). Gay male couples' attitudes toward using couples-based voluntary HIV counseling and testing. Arch Sex Behav.

[46] Sullivan PS, Peterson J, Rosenberg ES, Kelley CF, Cooper H, Vaughan A, Salazar LF, Frew P, Wingood G, Diclemente R, del Rio RC, Mulligan M, Sanchez TH (2014). Understanding racial HIV/STI disparities in black and white men who have sex with men: A multilevel approach. PLoS One.

[47] Lim JR, Sullivan PS, Salazar L, Spaulding AC, Dinenno EA (2011). History of arrest and associated factors among men who have sex with men. J Urban Health.

[48] Ford MA, Spicer CM, Committee to Review Data Systems for Monitoring HIV Care, Institute of Medicine of the National Academies (2012). Monitoring HIV Care in the United States: Indicators and Data Systems.

[49] McNutt LA, Gordon EJ, Uusküla A (2009). Informed recruitment in partner studies of HIV transmission: An ethical issue in couples research. BMC Med Ethics.

[50] Giordano TP, Guzman D, Clark R, Charlebois ED, Bangsberg DR (2004). Measuring adherence to antiretroviral therapy in a diverse population using a visual analogue scale. HIV Clin Trials.

[51] Salazar LF, Stephenson RB, Sullivan PS, Tarver R (2013). Development and validation of HIV-related dyadic measures for men who have sex with men. J Sex Res.

[52] Levinger G, Pietromonaco P (1989). Conflict Style Questionnaire.

[53] Heavey CL, Larson BM, Zumtobel DC, Christensen A (1996). The Communication Patterns Questionnaire: The reliability and validity of a constructive communication subscale. J Marriage Fam.

[54] Balfour L, Tasca GA, Kowal J, Corace K, Cooper CL, Angel JB, Garber G, Macpherson PA, Cameron DW (2007). Development and validation of the HIV Medication Readiness Scale. Assessment.

[55] Straus MA, Hamby SL, Boney-McCoy S, Sugarman DB (1996). The Revised Conflict Tactics Scales (CTS2): Development and preliminary psychometric data. J Fam Issues.

[56] (2008). SAS/STAT® 9.2 User’s Guide.

